# Claudin and transmembrane receptor protein gene expressions are reversely correlated in peritumoral brain edema

**DOI:** 10.1002/cam4.70111

**Published:** 2024-08-27

**Authors:** Anwar Abuelrub, Berkay Paker, Turker Kilic, Timucin Avsar

**Affiliations:** ^1^ Neuroscience Laboratory, Health Sciences Institute Bahcesehir University Istanbul Turkey; ^2^ Department of Neurosurgery Bahcesehir University School of Medicine Istanbul Turkey; ^3^ Department of Medical Biology Bahcesehir University School of Medicine Istanbul Turkey

**Keywords:** blood–brain barrier, claudin, glioma, IDH, peritumoral brain edema, transient receptor proteins

## Abstract

**Introduction:**

Peritumoral brain edema (PTBE) has been widely reported with many brain tumors, especially with glioma. Since the blood–brain barrier (BBB) is essential for maintaining minimal permeability, any alteration in the interaction of BBB components, specifically in astrocytes and tight junctions (TJ), can result in disrupting the homeostasis of the BBB and making it severely leaky, which subsequently generates edema.

**Objective:**

This study aimed to evaluate the functional gliovascular unit of the BBB by examining changes in the expression of claudin (CLDN) genes and the expression of transient receptor potential (TRP) membrane channels, additionally to define the correlation between their expressions. The evaluation was conducted using in vitro spheroid swelling models and tumor samples from glioma patients with PTBE.

**Results:**

The results of the spheroid model showed that the genes TRPC3, TRPC4, TRPC5, and TRPV1 were upregulated in glioma cells either wild‐type isocitrate dehydrogenase 1 (IDH1) or the IDH1 R132H mutant, with or without NaCl treatment. Furthermore, TRP genes appeared to adversely correlate with the up regulation of CLDN1, CLDN3, and CLDN5 genes. Besides, the upregulation of TRPC1 and TRPC4 in IDH1mt‐R132H glioma cells. On the other hand, the correlation analysis revealed different correlations between different proteins in PTBE. CLDN1 exhibits a slight positive correlation with CLDN3. Similarly, TRPV1 displays a slight positive correlation with TRPC1. In contrast, TRPC4 shows a slight negative correlation with TRPC5. On the other hand, TRPC3 demonstrates a slight positive correlation with TRPC5, while the non‐PTBE analysis highlights a moderate positive correlation between CLDN1 and TRPM4 while CLDN3 exhibits a moderate negative correlation with TRPC4. Additionally, CLDN5 demonstrates a slight negative correlation with TRPC4 but a moderate positive correlation with TRPC3. Furthermore, TRPC1 have a slight negative correlation with TRPV1, TRPC3 exhibiting a slight positive correlation with TRPC4, and TRPV1 showing a slight negative correlation with TRPC5.

**Conclusion:**

As a conclusion, the current study provided evidence of a slight negative correlation between TRPs and CLDN gene expression in PTBE patients and confirmatory results with some of the genes in cell model of edema.

## 1 INTRODUCTION

Peritumoral brain edema (PTBE) is one of the most prevalent glioma‐related conditions. Which is characterized mainly by disruption of the blood–brain barrier (BBB) and the frequent absence of tight junction (TJ) proteins. Furthermore, PTBE was linked with a number of risk factors, which hypothesized that the progression of malignancies is primarily associated with the presence of edema.^1^ Additionally, PTBE plays a key role in determining the glioma's clinical outcome,^2^ as the extent of edema can predict tumor migration activity as a result of the infiltration and invasion activity of glioblastoma multiforme (GBM), which are related to edema due to a compromised BBB.^3^ Last but not least, PTBE is considered life‐threatening due to its features that increase intracranial pressure, which could be fatal in many cases.^4^

The preservation of the homeostasis of the brain and minimal permeability depend on the BBB, where endothelial cells are tightly connected to each other via TJ, forming the neurovascular unit along with astrocytes and pericytes with neurons. Due to the sensitive nature of the BBB to changes in the surrounding microenvironment, such as trauma, variation in oxygen levels, neurodegenerative diseases, tumors, and inflammation,^5^ alterations in the interaction of neurovascular components can take place, especially in astrocytes and tight junction structure and function, which can cause BBB breakdown in tumor vasculature.^6^ Moreover, the astrocytes have the charge of maintaining the tight junction position; when TJ are compromised, the BBB is disrupted and becomes leaky, which allows the passage of hydrophilic molecules and pathogens to the brain parenchyma, resulting in hyperpermeability that eventually leads to edema.^7^

In the context of any ionic/metabolic imbalance, it could interfere with the expression of tight junction proteins, including claudin (CLDN), occluding, and zonula occluding. These proteins control the movement of solutes, ions, and other molecules between cells. For instance, an increase in intracellular sodium (Na^+^) levels can lead to cytotoxic or vasogenic edema, where fluid leaks out of the blood vessels and accumulates in the cells or extracellular spaces.^8^ Furthermore, glioblastoma cells can release more than 2000 different proteins into their surrounding microenvironment. The continuous interactions between endothelial cells and glioblastoma cells within this microenvironment lead to alterations in the structure and function of the endothelial cells. Consequently, the alterations in the properties of endothelial cells lead to the development of edema by enhancing the permeability of the BBB.^9^ One key regulator of BBB is the degree of tight junction‐associated CLDN proteins expressed which has a direct influence on the amount of paracellular fluid and ion flux, as well as charge selectivity. CLDN act as molecular gatekeepers, regulating the passage of substances between cells. Remarkably, the capillaries comprising the BBB exhibit the highest expression of CLDN proteins compared to any other tissue in the human body.^10^

On the other hand, the channels of TRP on the plasma membrane are characterized as cellular sensors for a variety of stimuli, both chemically and physically.^11^ They react with exceptional sensitivity to basic cellular signaling components such as temperature, voltage, osmotic changes, mechanical pressure, and ligand binding.^11^ TRP channels are activated by phospholipase C signaling pathways; PLC activation causes plasma membrane phosphatidyl inositol 4,5‐biphosphate PIP2 hydrolysis into diacylglycerol DAG and inositol 1,4,5‐triphosphate (IP3). These by‐products are essential second messengers that regulate a variety of cellular functions.^12^ Eventually, IP3 activates TRP channels. Thus, in response to either an increase in intracellular Ca^2+^ release from their internal cellular stores or activating Ca^2+^ inflow,^13^ generating a response from those channels is important to adapt to changes in the surrounding environment.^14^ Although a few research studies have demonstrated to investigate the pathological role of increased TRP channel expression in gliomas, they have reported a major role in regulating cell proliferation, apoptosis, differentiation, cancer progression, and metastasis. In addition, these proteins also control the BBB's endothelial and astrocyte cells' reactions to stress and changes in cell volume by increasing the influx of extracellular calcium channels.^15^, ^16^ The most prevalent TRP channels expressed in gliomas, identified as TRPC, TRPM, and TRPV, have revealed that the inflammation that follows any tumor to the brain causes BBB disruption and permeability to increase as a result of calcium movement inside the cell, where the levels of intracellular Ca^2+^ concentration increase and TJ are loose.^17^ Though the mechanism that controls the tightness and looseness of TJ remains unidentified.^18^

In general, the mechanism behind the edema formation associated with tumors is not well understood and not well addressed in previous research. After carefully reviewing the literature, it was found that CLDN and Transient Receptor Potential (TRP) membrane proteins exhibited major changes in their expression levels in edema. For this reason, our study focused on investigating the correlation between these two gene families in glioma, which is one of the most prominent cancers associated with edema formation. To perform this investigation, we will generate in vitro swollen spheroid models together with glioma PTBE patient samples in order to identify changes in the expression of paracellular CLDN and TRP. Furthermore, the use of isocitrate dehydrogenase 1 (IDH1) inhibitors is very limited, despite IDH1 being known as one of the most promising biomarkers for glioma diagnosis and prognosis.^19^ Therefore, it was also aimed to discover its effects on edema associated gene expression profile. The use of in vitro model is essential in this study, as it provides a controlled environment for accurately assessing gene expressions linked to edema. The spheroid models offer distinct advantages in validating the groundwork for further in vivo research as they closely mimic the in vivo microenvironment. Besides, in vitro model plays a critical role in confirming clinical sample findings under controlled conditions, serving as a platform for conducting proof‐of‐concept experiments. Long term goals of this study are to provide both gene expression pattern changes correlation in edema formation and proposing a new swollen in vitro model to understand therapeutic interventions of the target genes.

## 2 MATERIALS AND METHODS

**FIGURE 1:**
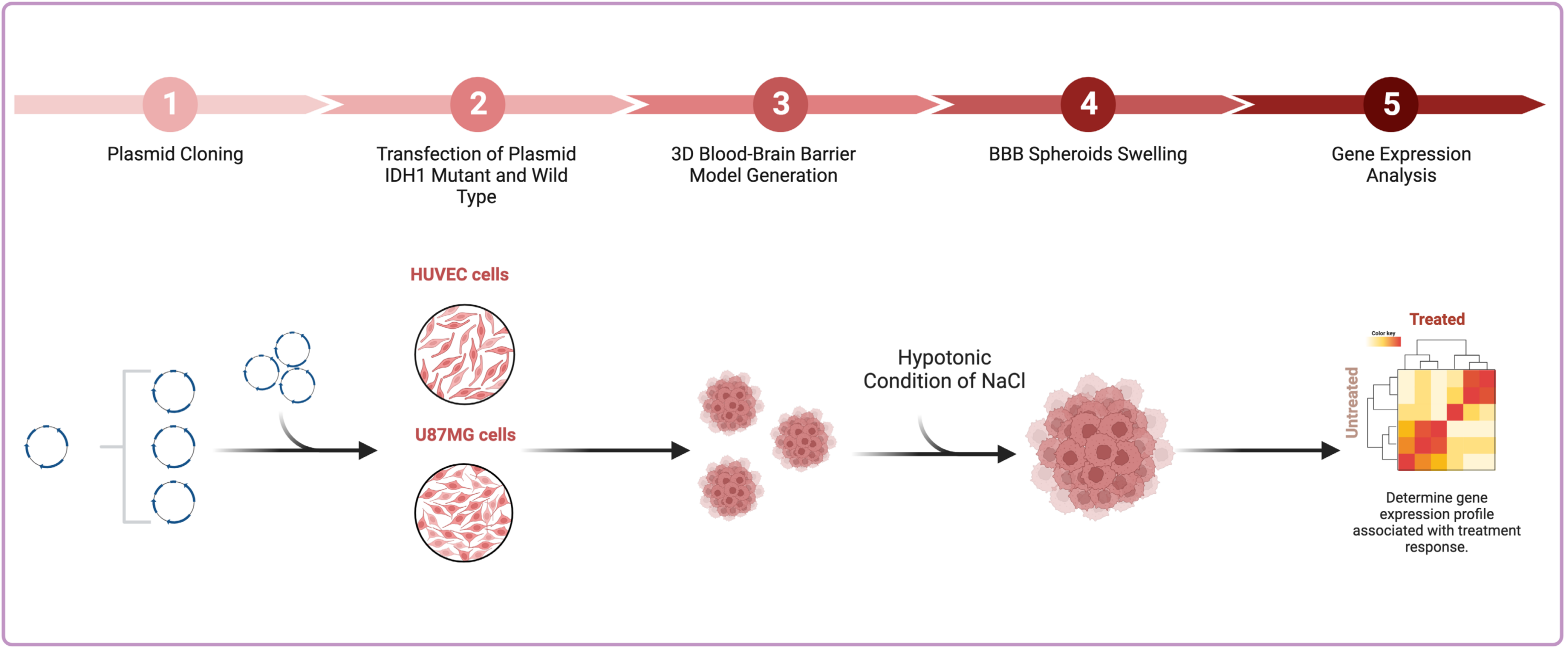
Schematic overview of the study workflow for a 3D spheroid model of the blood–brain barrier.

Graphical abstract of in vitro studies was provided (Figure 1).

### 2.1 Preparation of cell lines

Respectively, we employed Human umbilical vein endothelial cells (HUVECs) and, U87MG cells that derived from a human GBM to generate a spheroid model system to investigate the BBB associated gene expressions. Both of these cells have been widely used in simulating elements of the tumor microenvironment and the BBB interface in many research studies pertaining to glioma‐related disorders, such as PTBE.^20^, ^21^, ^22^ Furthermore, to investigate the role of IDH1 expression either in wildtype (IDH1wt) or mutant (IDH1mt‐R132H) and IDH1mt‐R132H Inhibitor (IDH1mt‐R132H + IDH1Inh‐R132H) form in U87MG and HUVEC co‐cultured spheroid models in vitro, plasmid vector containing IDH1 gene was introduced in the cells to increase the expression of R132H by using pcDNA3‐Flag‐IDH1 plasmid (Cat. No. 62906) that was obtained from Add Gene Inc. (Cambridge, MA). After plasmid cultivation and isolation steps, cells were transiently transfected with the lipofectamine 3000 transfection kit (L3000008; thermo‐fisher scientific): 1 × 10^5^ cells were seeded and incubated for 24 h at 37°C and 5% CO_2_. On the next day, the media of the cells were replaced with fresh, complete DMEM supplemented with 10% fetal bovine serum and 1% antibiotic/anti‐miotic. The DNA/lipid complex was prepared in a medium‐free antibiotic to prevent cell death with a 1:2:2 ratio of DNA:lipo3000:p3000 reagent. After incubation for 15 min, the plasmid‐lipofectamine complex was distributed drop by drop over the cells. After 3 days, the transfection efficiency of U87MG and HUVEC cells was assessed by a fluorescent microscope. Cells were counted on the image as a ratio of transfected fluorescent cells over a total number of cells to evaluate the efficiency of the transfection. Furthermore, IDH1mt‐R132H + IDH1Inh‐R132H group cells were treated with 20 μM of an IDH1‐inhibitor, ML‐309 (Sigma Aldrich, SML 1298)^23^ was prepared in 1 mL of complete DMEM.

### 2.2 In‐Vitro BBB spheroid 3D model

For the BBB 3D structural model, co‐cultured spheroids containing the human glioma U87MG cell line and HUVEC were used. To prepare the spheroids, cell lines were released by trypsin/EDTA and resuspended in a neurobasal medium supplemented with 1× L‐glutamine, 1× B27, and 1× N2 supplements. Twenty nanograms per milliliter of basic fibroblast growth factor and 20 ng/mL of epidermal growth factor. On the first day, 20 μM of a BCL‐2 (243) inhibitor was added to the medium. The number of each cell line was determined using a hematocytometer: 1 × 10^5^ of each cell type were seeded onto a T‐25 low attachment cell culture flask with a 1:1 ratio (final volume = 5 mL). Further, cells were placed in a humidified incubator at 37°C with 5% carbon dioxide for 10 days to allow the assembly of multicellular BBB spheroids. During those 10 days, the medium and growth factors were refreshed every 3 days. Before the necrotic core is developed, spheroids are collected in a 15 mL falcon by centrifuging at 100 × g for 5 min.

### 2.3 Inducing cell swelling under hypotonic conditions

A range of different Sodium Chloride (NaCl) concentrations between 0.001M and 0.009M were examined in order to identify the best concentration of NaCl for stimulating cells to swell. The pH of the solutions was adjusted to 7.4. In order to induce cell swelling, a mixture of 1 × 10^5^ U87MG and HUVEC cells was cultured in 35‐mm dishes and treated for 10 min at room temperature in 1 mL of NaCl at various concentrations. The changes in cell volume were detected after 10 minutes of exposure to each solution with immunofluorescence staining. The control group was incubated in complete DMEM, free of NaCl.

### 2.4 Immunofluorescence staining

To visualize the cells following swelling by Immunofluorescence (IF) staining. First, cells were fixed for 20 min at room temperature with 3% PFA (in PBS pH 7.4), then washed 3× for 5 min with PBS, followed by 30 mM glycine incubation for 5 min to block non‐specific binding, and then washed with 2× PBS. To increase the permeability of antibodies, 0.1% Triton X‐100 (in PBS) was used for 10 min. After the permeabilization step, the washing step was repeated 3× for 5 min, and then cells were incubated in the primary beta tubulin mouse monoclonal antibody (MA5‐16308) in PBS for 30 min at room temperature. Followed by 3× PBS washing steps for 5 min each and the secondary antibody incubation of Alexa Fluor goat anti‐mouse (1:1000; Invitrogen) that was diluted in PBS for 30 min at room temperature, with the washing cells step repeated 3×. Finally, the slides were mounted and analyzed under the fluorescence microscope.

### 2.5 MTT assay

For validating further experiments within the scope of no variations in cell viability. Cells were assessed by MTT under hypotonic conditions. In a 96‐well plate, the viability of spheroids under NaCl hypotonic conditions was compared with that of untreated spheroids to determine the optimal cell viability. After 10 min of exposure to hypotonic solutions, the spheroids were incubated for 3 h with 10 μL of 5 mg/mL MTT solution added to each well. One hundred microliter of MTT solubilization solution was added to each well and incubated for 15 min to solubilize formazan crystals. In the end, the absorbance at 570 nm was measured with a Hidex Multimode Microplate Reader.

### 2.6 Gene expression

To quantify the gene expression of tight junction (TJ) CLDN and TRPs' with the primers mentioned in Table S1 by using real‐time qPCR in the patients and a 3D BBB model. Total RNA isolation was performed using a Primezol RNA isolation kit by following the manufacturer's instructions. The isolated RNA quality was validated by nanodrop (Nabi) and agarose gel running to confirm its integrity after the extraction. One hundred nanogram of isolated RNA was reversed into cDNA by using a cDNA synthesis kit with RNase inhibitor (A.B.T.). Consequently, qRT‐PCR was performed with Bio‐Rad's SSO advanced universal SYBR green supermix. The cycle threshold genes were normalized with GAPDH.

### 2.7 Patients and samples

Permission to review patients' medical records was obtained from Medical Park Hospital in Istanbul, Turkey. Between August 2020 and November 2021, a total of 60 patients with a histopathological diagnosis of grade I, II, III, and IV gliomas were included. The specific features of patients are listed in Table 1. In this study, the patients were classified according to the presence of edema into PTBE and non‐PTBE groups. Patients who showed a clear image of PTBE confirmed by a pathologist were included in the PTBE group regardless of glioma grade.

**TABLE 1:** Classification of patients according to gender, grade, and disease phenotype.

Characteristic	Cohort		
	High grade glioma	Low grade glioma	Total
Gender			
Female	14	8	22
Male	21	17	38
IDH			
Mutant	11	25	36
Wild type	22	2	24
Peritumoral brain edema			
PTBE	30	3	33
Non‐PTBE	6	21	27

### 2.8 Statistical analyses

All statistical analyses were performed using GraphPad Prism 9. The one‐way ANOVA was used to analyze patients and in vitro studies. Correlation analysis was done by using one‐tail Pearson correlation test. A *p* value of 0.05 was regarded as statistically significant for all comparisons when significant *p* values were found.

## 3 RESULTS

### 3.1 Immunofluorescence staining demonstrated a cell size increase by NaCl hypotonic solution

IF staining revealed the role of NaCl‐induced volume changes in a mix of U87MG and HUVEC cells. The staining was performed using a beta‐tubulin antibody. In which an increase in the cell volume from an average size of 250 μm and swelling to increase the size to an average of 750 μm are induced by both 0.002% and 0.004% NaCl hypotonic solutions as illustrated in Figure S1. Suggesting that these conditions could be applied to co‐cultured U87MG and HUVEC cells to generate the cell swelling of a spheroid model.

### 3.2 Cell swelling was generated in an in vitro blood–brain barrier spheroid model

**FIGURE 2:**
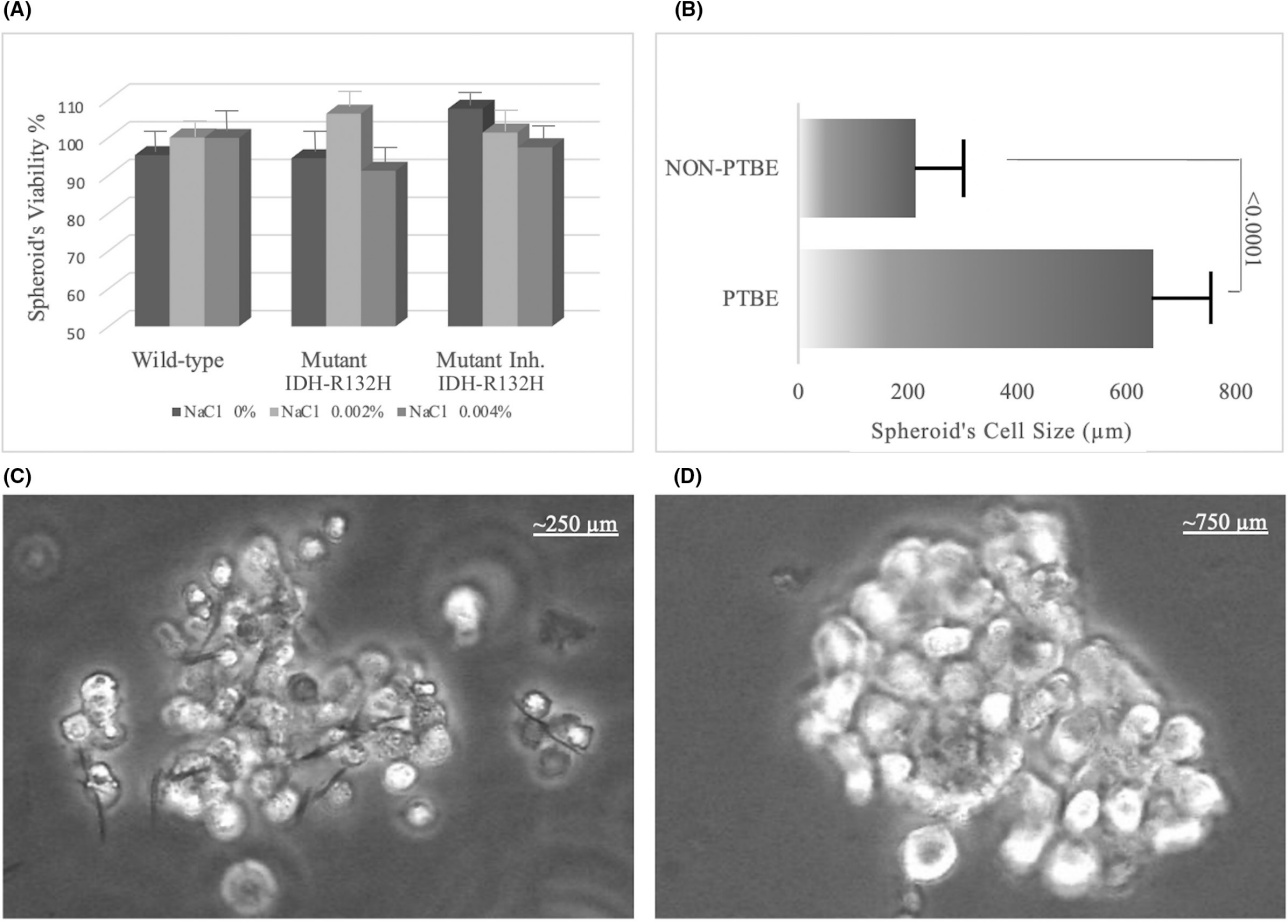
(A) The viability investigation of transfected cells with hypotonic treatment showed no significant change in their values, which validates the co‐cultured spheroids as a model for the blood–brain barrier (BBB) in vitro. The effect of subjecting a spheroid model to hypotonic salt concentration (0.002%) denotes a relative gain of extracellular fluids, leading to a significant increase in the diameter of the cell size of spheroids. (B) Disparities in the diameter of cells between the control group (NaCl‐free) ~250 μm (C) and the treated groups with 0.002% NaCl ~750 μm (D) error bars indicate the standard deviation (*n* = 3).

A comprehensive microscopic examination of the cell–cell interaction and the gliovascular unit between U87MG and HUVEC cells in the spheroid model indicates a highly formed tight junction that acts as a part of the spheroid barrier. Upon hypotonic simulation of the co‐cultured HUVEC and U87MG cells, cell swelling was generated in 10 min with a 0.002% NaCl solution (Figure 2). These data indicated that the hypotonic conditions enhanced the swelling in the BBB spheroid model. To validate the optimized conditions of the hypotonic solutions on the viability of the cells and to carry out experiments with no significant change in cell viability. The MTT assay was performed to make sure the proliferation of co‐cultured spheroids would not change under transfection or hypotonic solutions. Clearly, the experimental results in Figure 2 showed no noted change in cell viability between the groups. NaCl hypotonic 0.002% would be used for further experiments in the quantitative evaluation to induce swelling, as it showed earlier the most significant increase in the cell size.

### 3.3 Dysregulation of CLDN and TRP gene expressions in a swelled spheroid model

**FIGURE 3:**
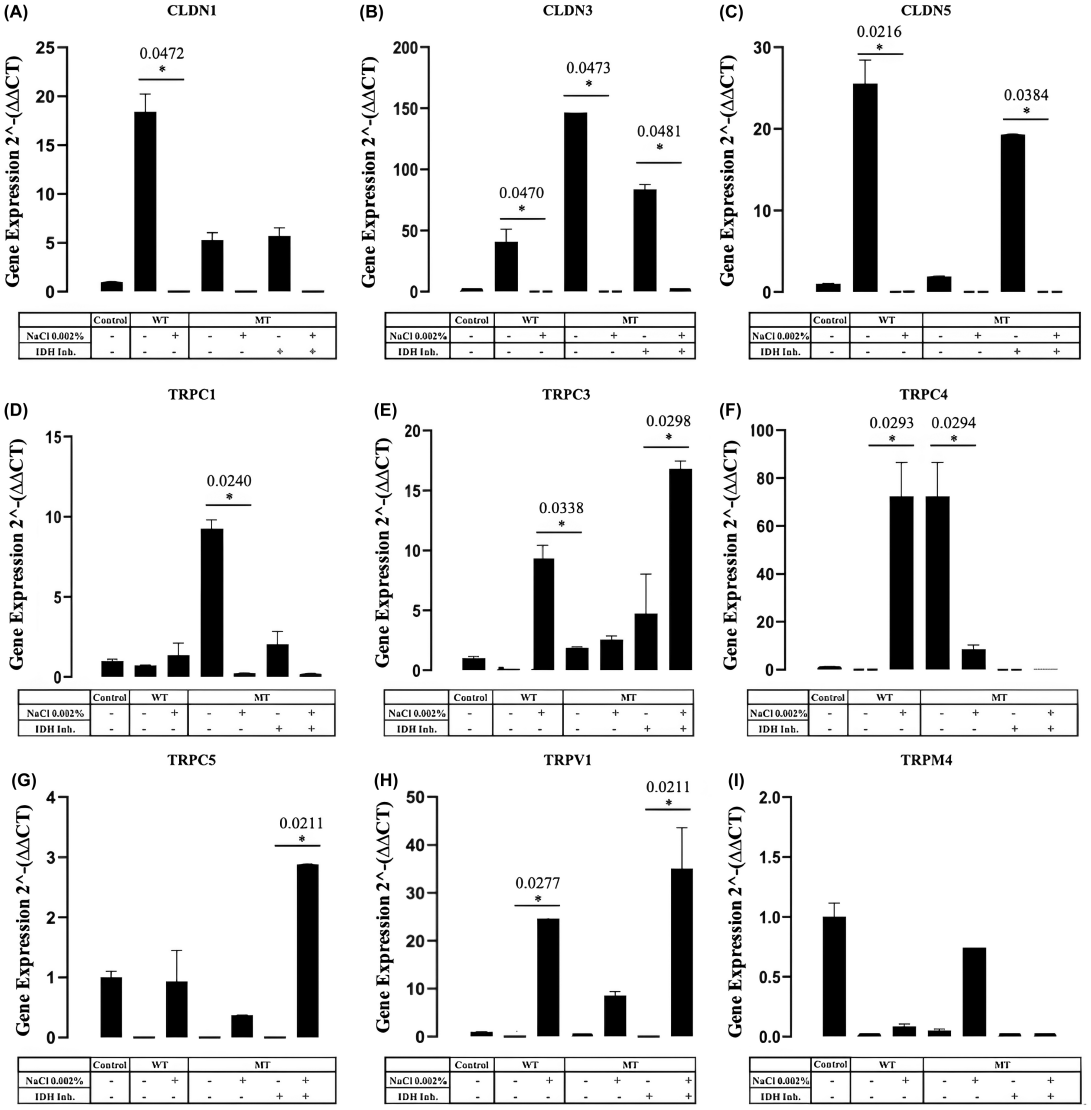
(A) A noticeable increase in CLDN1 expression has been observed in transfected IDH1wt spheroids (*p 0.0472) in the absence of 0.002% NaCl. (B) A major increase in the expression of CLDN3 in the absence of 0.002% NaCl in transfected spheroids (IDH1wt *p 0.0470, IDH1mt‐R132H *p 0.0473, IDH1mt‐R132H + IDH Inh‐R132H *p 0.0481). (C) The expression of CLDN5 greatly increased in the absence of 0.002% NaCl (IDH1wt *p 0.0216, and IDH1mt‐R132H + IDH Inh‐R132H *p 0.0384). (D) In the absence of 0.002% NaCl, TRPC1 expression significantly increased (IDH1mt‐R132H *p 0.0240). (E) TRPC3 expression significantly increased in the presence of 0.002% NaCl (IDH1wt *p 0.0338 and IDH1mt‐R132H + IDH Inh‐R132H *p 0.298). (F) Both IDH1wt (0.002% NaCl) and IDH1mt‐R132H (NaCl‐free) TRPC4 expression widely increased (*p 0.0293, *p 0.0294). (G) In the presence of 0.002% NaCl, the expression of TRPC5 increases in the IDH1mt‐R132H + IDH Inh‐R132H *p 0.0211. (H) In the presence of 0.002% NaCl, there is a notable increase in the expression of TRPV1 (IDH1wt *p 0.0277, and IDH1mt‐R132H + IDH Inh‐R132H *p 0.0211). (I) TRPM4 gene expression did not significantly change under any condition. Error bars indicate the standard deviation (*n* = 3).

The spheroid model was used to mimic edema formation in in vitro conditions to evaluate edema associated genes' expression. The constitutive correlation of CLDNs in TJ that regulate paracellular pathway permeability and TRP that regulates transcellular permeability was tested on a spheroid model. Results indicated dysregulation of TJ CLDN and membrane TRP's gene expressions in co‐cultured BBB spheroids following the treatment of NaCl in a dose‐ and time‐dependent manner that resulted in swelling. Whereas CLDN1 gene expressions in IDH1wt showed a significant upregulation (*p = 0.0472), IDH1mt‐R132H and IDH1mt‐R132H + IDH1Inh‐R132H showed a slight increase in their expressions however they were not significant. Opposite to that, Na treatment (NaCltr) IDH1wt, IDH1mt‐R132H, and IDH1mt‐R132H + IDH1Inh‐R132H showed no significant change in their expressions (Figure 3A). Moreover, a major increase was observed as well in the expressions of CLDN3 of IDH1wt (*p = 0.0470), IDH1mt‐R132H (*p = 0.0473), and IDH1mt‐R132H + IDH1Inh (*p = 0.0481) with free NaCltr. In contrast to NaCltr IDH1wt, IDH1mt‐R132H, and IDH1mt‐R132H + IDH1Inh‐R132H that showed free change in their expressions (Figure 3B). In Figure 3C the expression of CLDN5 greatly increased in free NaCltr IDH1wt (*p = 0.0216) and IDH1mt‐R132H + IDH1Inh‐R132H (*p = 0.0384), while other treated groups showed no significant change. Besides, the increased expression of TRPC1in IDH1mt‐R132H with free NaCltr (*p = 0.0240) while other groups had no change (Figure 3D). The effect on IDH1 R132H inhibitor was also found to be decreasing the CLDN family genes however, transient receptor channel associated genes have not been significantly affected upon inhibitor treatment.

Additionally, TRPC3 expression significantly increased in NaCltr of IDH1wt (*p = 0.0338) and IDH1mt‐R132H + IDH1Inh‐R132H (*p = 0.298) on the other hand the other groups showed no statistical change as illustrated in Figure 3E. Furthermore, in Figure 3F TRPC4 expressions for both NaCltr IDH1wt (*p = 0.0293) and IDH1mt‐R132H (*p = 0.0294) widely increased. Along with that (Figure 3G) in the presence of NaCltr, the expression of TRPC5 increases in the IDH1mt‐R132H + IDH1Inh‐R132H (*p = 0.0211) while free NaCltr IDH1wt, IDH1mt‐R132H, and IDH1mt‐R132H + IDH1Inh‐R132H showed no significant change in their expressions. Figure 3H shows a notable increase of the expressions of TRPV1 in NaCltr groups for both IDH1wt (*p = 0.0277), IDH1mt‐R132H + IDH1Inh‐R132H (*p = 0.0211) with no significant change in other groups. Finally, TRPM4 gene expression did not significantly change under any condition.

### 3.4 Alteration of CLDN and TRPs gene expressions in the presence of PTBE in patient samples

**FIGURE 4:**
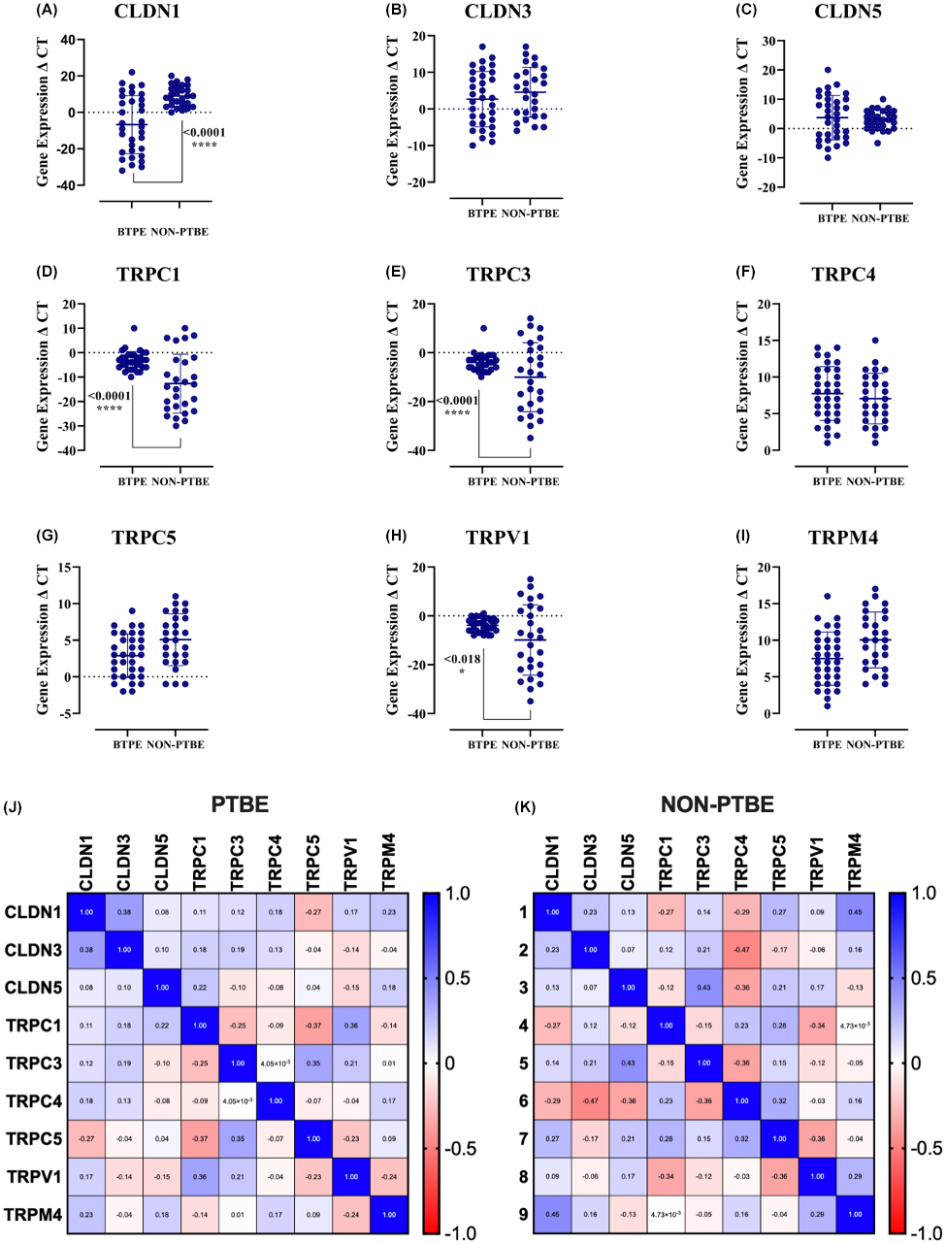
We analyzed six gap junction paracellular pathway‐responsible genes and three transcellular membrane genes in 60 glioma patients. CLDN and TRP have regulatory roles in PTBE in gliomas. Evaluating the expressions of these genes by quantitative RT‐PCR evidenced a marked variation in CLDN1 mRNA levels; in Peritumoral brain edema (PTBE) patients, the expression was significantly higher than patients with no PTBE (****p < 0.0001) (A), while the expression of TRPC1, TRPC3, and TRPV1 was significantly lower in PTBE patients compared to non‐PTBE patients (****p < 0.0001) (D, E, H). On the other hand, no gene expression differences in (B, C, F, G and I) were observed between PTBE and non‐PTBE pateints; conversely, (J) the heatmap of PTBE patients showed a positive correlation between TRP family genes specifically (TRPC1, TRPC3, TRPC4, TRPV1, TRPM4); (K) the non‐PTBE heatmap showed a positive correlation between the CLDN gene family, besides TRPC4 and TRPC5.

The expression of all genes showed variations in both PTBE and non‐PTBE patients' tumors. In contrast to non‐PTBE controls, patients with PTBE had lower gene expression for CLDN1 (****p < 0.0001) (Figure 4A). While the expression of CLDN3 Figure 4B showed no difference between PTBE and non‐PTBE groups, alongside CLDN5 expressions, there were no statistically significant differences between the PTBE group and non‐PTBE patients (Figure 4C). On the other hand, TRPC1 in Figure 4D reported a significant downregulation in their expressions in PTBE patients compared to non‐PTBE patients (****p < 0.0001). Conversely, (Figure 4E) TRPC3 reported a significant downregulation in their expressions in PTBE patients compared to non‐PTBE patients (****p < 0.0001). The expressions of TRPC4 showed no statistically significant differences between the PTBE group and non‐PTBE patients (Figure 4F). In addition to the expressions of TRPC5, no significant changes were reported between the PTBE group and non‐PTBE patients (Figure 4G). Meanwhile, TRPV1 showed a significant downregulation in PTBE patients compared to non‐PTBE patients (****p < 0.0001), as indicated in Figure 4H. Further, the expressions of TRPM4 demonstrated no significant change between the PTBE group and non‐PTBE patients. The heatmap on the left labeled PTBE illustrates several correlations. CLDN1 exhibits a slight positive correlation (0.38) with CLDN3 (*p = 0.015). Similarly, TRPV1 displays a slight positive correlation (0.36) with TRPC1 (*p = 0.02). While TRPC4 shows a slight negative correlation with TRPC5 (−0.37). Conversely, TRPC3 demonstrates a slight positive correlation with TRPC5 (0.35) (*p = 0.024). In Figure 4K, there is a moderate positive correlation between CLDN1 and TRPM4 (0.45) (***p = 0.009), whereas CLDN3 exhibits a moderate negative correlation with TRPC4 (−0.47) (***p = 0.007). Additionally, CLDN5 demonstrates a slight negative correlation with TRPC4 (−0.36) (*p = 0.034) but a moderate positive correlation with TRPC3 (0.43) (*p = 0.012). TRPC1 shows a slight negative correlation with TRPV1 (−0.34) (*p = 0.039), TRPC3 has a slight positive correlation with TRPC4 (0.32) (*p = 0.032), and TRPV1 exhibits a slight negative correlation with TRPC5 (−0.36) (*p = 0.032).

## 4 DISCUSSION

This study investigated how CLDN and TRP gene expressions change in the gliovascular unit of a disrupted BBB in one of the most common glioma‐related complications, PTBE. Predominantly under normal and pathological conditions, TJ compartments contribute to the expression and localization of the BBB as well as to the tightness of the BBB.^24^ In this study, it is hypothesized that CLDN and TRP gene expression are reversely dysregulated in PTBE formation and associated in vitro spheroid models.

The majority of 3D spheroid models of the BBB in vitro contain all major cell types, including endothelial cells and astrocytes, to closely mimic normal human brain tissue. The spheroids exhibit the expression of cell‐specific markers of adherent junctions, TJ, and adherent junction‐associated proteins.^25^ In this study, spheroid model has been used to evaluate the effect of BBB disruption on edema formation. Furthermore, to induce cell swelling by changing the tonicity of the cell medium, which will result in either isotonic, hypertonic, or hypotonic conditions, which is associated with movement of fluids into and/or out of the cell. The change in a cell's volume depends on the solution surrounding the cell and whether the cell membrane allows solutes to pass through or not. This is due to a process called osmosis.^26^ It is necessary to use the additional term tonicity if a membrane is not equally permeable to all solutes since this will cause a change in the way water moves when a low osmolarity hypotonic solution of NaCl is exposed to the cells with higher water concentrations and a lower effective osmotic pressure outside the cell than the internal fluid, causing fluids to undergo net permeability via osmosis inside the cells, leading to cell swelling.^26^ In this study, treatment with NaCl hypotonic solution resulted in a cell size increase from normal size ~250 to ~750 μm which is considered as cell swelling.

**FIGURE 5:**
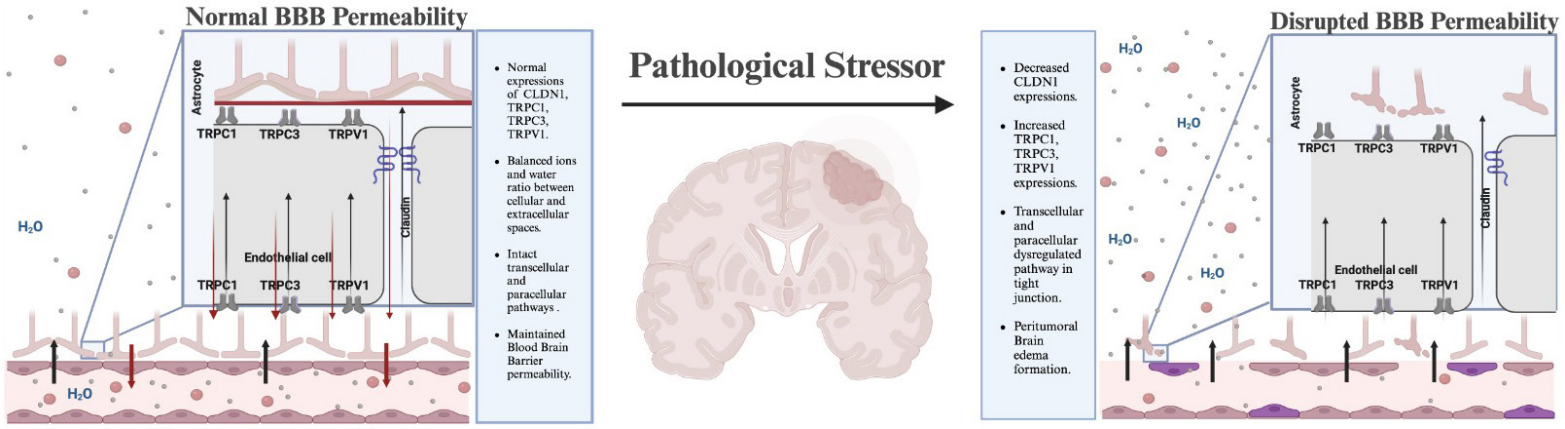
The BBB reversibly expresses claudin (CLDN) and transient receptor channels in astrocytes and endothelial cells in gliovascular units, which significantly increases permeability in cases of peritumoral brain edema. Where the absence and low expression of CLDN in TJ and increased transient receptor channel expression in astrocytes result in excess paracellular and intracellular movement of solutes, nanoparticles, and fluid accumulation.

Previous investigations have demonstrated TJ proteins' role in the maintenance of the tightness of the BBB, such as the dominant component of the tight junction, CLDN, which has shown a significant regulator property of the paracellular permeability of the tight junction.^8^ The absence and low expression of TJ proteins play a role in edema formation and development and could influence the outcome in many pathological cases, such as stroke and TBI cases.^8^, ^27^ According to the findings of a prior glioma investigation, there may be a connection between the reduction of CLDN 1 and changes in tight junction morphology, which are likely to be related to an increase in endothelial permeability. In order to highlight how undifferentiated tumor micro‐vessels are, the data point to a dysregulation of junctional proteins as the cause of the increased microvascular permeability in human gliomas, which contributes to the clinically severe symptoms of brain edema.^28^ A few studies have shown that elevated TRP channel expression in gliomas has a pathogenic effect; these channels affect the development and spread of cancer as well as cellular differentiation, proliferation, and death.^29^ As TRP proteins have a role in the endothelial BBB cells, especially in stressful events, they cause changes in cell volume and BBB permeability by causing an influx of calcium channels outside of cells.^15^, ^16^ The most frequently expressed TRP channels are TRPC, TRPM, and TRPV in gliomas that have been found so far.^17^ We reported a negative correlation between downregulated CLDN1 and upregulated TRPC1, TRPC3, and TRPV1 gene expressions in PTBE. Overall, considering the previous results and our findings, we propose that these three genes have pivotal role in edema formation (Figure 5).

One of the Ca^2+^‐mediated channels is the TRP channel, which is regulated by inositol 1,4,5 triphosphate receptors (IP3R) on the endoplasmic reticulum (ER). Many studies have confirmed that changes in the extracellular or intracellular calcium concentration that are regulated by inositol 1,4,5 IP3 disrupt the components of TJ.^30^, ^31^ TRP channels have been shown to contribute to edema formation and progression in many pathological conditions, such as respiratory diseases, both cytotoxic and vasogenic edema after traumatic brain injury (TBI), and ischemia.^32^, ^33^, ^34^, ^35^, ^36^ Under physiological circumstances, BBB TJs' CLDNs sustains barrier function in correlation to each other, and disruption of their localization due to external or internal factors at the TJs can lead to barrier breakdown due to their low expression.^37^ Our findings showed a slight negative relationship between CLDN and TRP families, with down‐regulation of CLDN1 and up‐regulation of TRPC1, TRPC3, and TRPV1 in PTBE patients.

These findings suggest that dysregulation of both CLDN and TRP gene expressions may contribute to the disruption of BBB integrity, leading to PTBE formation. This study provides evidence of a negative correlation between TRP and CLDN gene expression in PTBE patients and confirms these results with some genes in a cell model of edema. The dysregulation of these genes suggests their involvement in the pathogenesis of PTBE‐associated gliomas. Understanding the molecular mechanisms underlying PTBE formation could lead to the development of targeted therapies aimed at preserving BBB integrity and reducing edema in glioma patients. However, further studies are needed to elucidate the comprehensive roles of CLDN and TRP genes in PTBE pathogenesis and to explore potential therapeutic interventions based on these findings.

## 5 CONCLUSION

This study indicated a slight negative regulatory relationship between elevated TRP channels genes and reduced CLDNs genes by using tumors with edema and in vitro spheroid model of edema. We propose that TRP channel and CLDN family genes have pivotal role in edema formation in brain tumors.

## 6 STUDY LIMITATIONS

Although the tumor microenvironment was modeled using an in vitro spheroid model in this study, it might not accurately reflect the complexities of the BBB. More thorough insights might be obtained via in vivo investigations. The study effectively analyzed gene expression patterns; however, there is still a need to explore the functional implications of the identified dysregulations. Acknowledging this, it becomes evident that further investigation into the functional roles of TRP and CLDN proteins in peritumoral edema progression could significantly enhance the understanding of their dysregulations. By integrating functional experimental methods with gene expression analysis, future research can provide promising insights for further understanding of the mechanism. Furthermore, the narrow focus on specific CLDN and TRP genes related to the BBB limited the scope. Broadening the analysis to include other relevant tight junction components, pathways, and molecules involved in BBA integrity and tumor pathogenesis could lead to a more holistic understanding in addition to the opportunity to identify more potential effective therapeutic interventions.

## AUTHOR CONTRIBUTIONS

**Anwar Abuelrub:** Conceptualization (lead); data curation (lead); formal analysis (lead); investigation (lead); methodology (lead); project administration (lead); validation (lead); visualization (lead); writing – original draft (lead); writing – review and editing (equal). **Berkay Paker:** Resources (equal). **Turker Kilic:** Resources (equal). **Timucin Avsar:** Supervision (equal); writing – review and editing (equal).

## FUNDING INFORMATION

This research did not receive any specific grant from funding agencies in the public, commercial, or not‐for‐profit sectors.

## CONFLICT OF INTEREST STATEMENT

The authors declare no conflict of interest.

## ETHICS STATEMENT

The study was approved by Bahcesehir University institutional ethics board.

## CONSENT

The study was performed after written informed consent was obtained from all patients enrolled in the study. The scientific ethical committee approved the collection and usage of tumor (August 2020 and November 2021).

## Supporting information


Data S1:

## Data Availability

Data will be made available on request.
