# Supplementary material for: Claudin and transmembrane receptor protein gene expressions are reversely correlated in peritumoral brain edema
Source: Cancer Med. 2024 Aug 27;13(16):e70111. doi: 10.1002/cam4.70111 (PMC11347986; doi:10.1002/cam4.70111)
Supplement: Supplementary file 1 — Data S1: [file CAM4-13-e70111-s001.docx]

# Supplementary Data

Table 1. Primer’s sequence

| **Primer** | **Forward** | **Reverse** |
| --- | --- | --- |
| GAPDH | 5’-TGCACCACCAACTGCTTAGC-3’ | 5’-GGCATGGACTGTGGTCATGAG-3’ |
| CLDN1 | 5’-TCATTGGGGGTGCGATATTT-3’ | 5’-TTGGTGTTGGGTAAGAGGTT-3’ |
| CLDN3 | 5’-CACCATTATCCGGGACTTCT-3’ | 5’-TGTCCCTTAGACGTAGTCCT-3’ |
| CLDN5 | 5’-AGATTGAGAGGTCTGGGAAG-3’ | 5’-CAGCACTGTCTCTCTCATCC-3’ |
| TRPC1 | 5’-CTACAGAGGAGCAGAGGATG-3’ | 5’-CCAGGAAGAGGACGAGAGAT-3’ |
| TRPC3 | 5’-GGGTAACTCAAAGTCCAGGT-3’ | 5’-TGATAACGTGTTGGCTGATTG-3’ |
| TRPC4 | 5’-TGTCTTCTCTGTGTGCTACC-3’ | 5’-GACCCACGGTAATATCATCCA-3’ |
| TRPC5 | 5’-CCCATCTTTGTCTCTAGCCTG-3’ | 5’-TCTTGCCAAATGGGTCTTGAT-3’ |
| TRPV1 | 5’-TGACAGTGTGATGGAGAGT-3’ | 5’-TCTGTGCTGCTCCATTTCTT-3’ |
| TRPM4 | 5’-CAAGAAGAAGACCTGCACGA-3’ | 5’-TCCGTGGTGTGTGCATC-3’ |

#
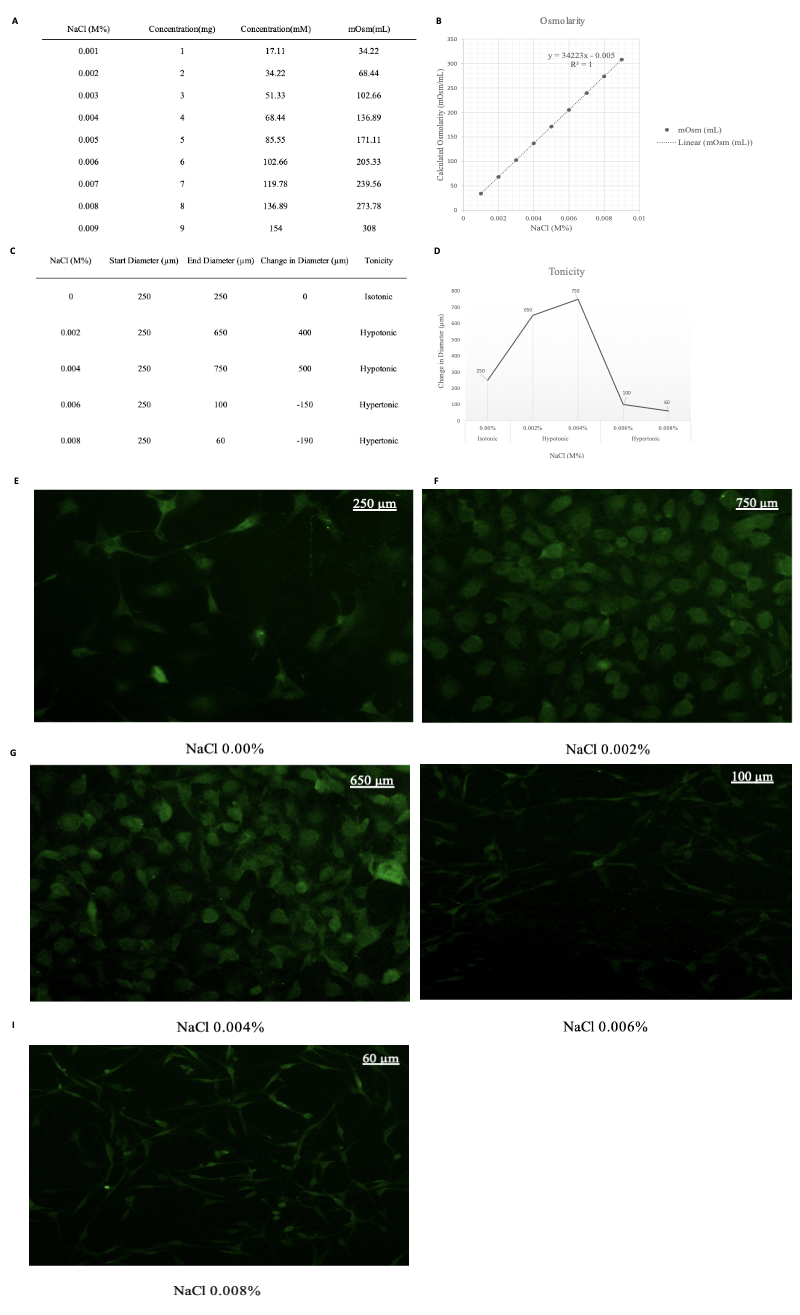


Figure 2. Contribution of different NaCl concentrations to the osmolarity (**A,B**) The calculations showed that higher concentrations of NaCl aligned with higher osmolarity rates, such as NaCl of 0.001% mM (34.22 mOsm/mL) and 0.009% NaCl (308 mOsm/mL). (**C,D**) Schematically illustrates the regulatory effect of NaCl concentration on tonicity; respectively, the rates of tonicity drop with greater concentrations of NaCl. The effect of NaCl concentration on cell size (**F,G**) These values showed significant cell diameter increases compared to the control group (NaCl-free) with hypotonic NaCl solutions (0.002% NaCl ****P <0.0001 and 0.004% NaCl *P <0.05), and hypertonic solutions with (**H,I**) NaCl (0.006%, 0.008%) decreased the cell diameter significantly ****P <0.0001.

Table 2: P value of the non-PTBE correlation

|  | **CLDN1** | **CLDN3** | **CLDN5** | **TRPC1** | **TRPC3** | **TRPC4** | **TRPC5** | **TRPV1** | **TRPM4** |
| --- | --- | --- | --- | --- | --- | --- | --- | --- | --- |
| **CLDN1** |  | 0.129 | 0.266 | 0.089 | 0.236 | 0.069 | 0.089 | 0.322 | 0.009 |
| **CLDN3** | 0.129 |  | 0.357 | 0.278 | 0.150 | 0.007 | 0.201 | 0.393 | 0.211 |
| **CLDN5** | 0.266 | 0.357 |  | 0.268 | 0.012 | 0.034 | 0.143 | 0.202 | 0.262 |
| **TRPC1** | 0.089 | 0.278 | 0.268 |  | 0.224 | 0.127 | 0.075 | 0.039 | 0.491 |
| **TRPC3** | 0.236 | 0.150 | 0.012 | 0.224 |  | 0.032 | 0.233 | 0.271 | 0.397 |
| **TRPC4** | 0.069 | 0.007 | 0.034 | 0.127 | 0.032 |  | 0.052 | 0.434 | 0.208 |
| **TRPC5** | 0.089 | 0.201 | 0.143 | 0.075 | 0.233 | 0.052 |  | 0.032 | 0.416 |
| **TRPV1** | 0.322 | 0.393 | 0.202 | 0.039 | 0.271 | 0.434 | 0.032 |  | 0.071 |
| **TRPM4** | 0.009 | 0.211 | 0.262 | 0.491 | 0.397 | 0.208 | 0.416 | 0.071 |  |

Table 3: Table 2: P value of the PTBE correlation

|  | **CLDN1** | **CLDN3** | **CLDN5** | **TRPC1** | **TRPC3** | **TRPC4** | **TRPC5** | **TRPV1** | **TRPM4** |
| --- | --- | --- | --- | --- | --- | --- | --- | --- | --- |
| **CLDN1** |  | 0.015 | 0.333 | 0.280 | 0.249 | 0.158 | 0.066 | 0.166 | 0.102 |
| **CLDN3** | 0.015 |  | 0.299 | 0.160 | 0.149 | 0.235 | 0.402 | 0.224 | 0.418 |
| **CLDN5** | 0.333 | 0.299 |  | 0.113 | 0.293 | 0.338 | 0.407 | 0.202 | 0.152 |
| **TRPC1** | 0.280 | 0.160 | 0.113 |  | 0.076 | 0.316 | 0.017 | 0.020 | 0.223 |
| **TRPC3** | 0.249 | 0.149 | 0.293 | 0.076 |  | 0.491 | 0.024 | 0.118 | 0.482 |
| **TRPC4** | 0.158 | 0.235 | 0.338 | 0.316 | 0.491 |  | 0.352 | 0.421 | 0.173 |
| **TRPC5** | 0.066 | 0.402 | 0.407 | 0.017 | 0.024 | 0.352 |  | 0.100 | 0.316 |
| **TRPV1** | 0.166 | 0.224 | 0.202 | 0.020 | 0.118 | 0.421 | 0.100 |  | 0.088 |
| **TRPM4** | 0.102 | 0.418 | 0.152 | 0.223 | 0.482 | 0.173 | 0.316 | 0.088 |  |
